# Evaluating a scale of excessive mind wandering among males and females with and without attention-deficit/hyperactivity disorder from a population sample

**DOI:** 10.1038/s41598-019-39227-w

**Published:** 2019-02-28

**Authors:** Florence D. Mowlem, Jessica Agnew-Blais, Jean-Baptiste Pingault, Philip Asherson

**Affiliations:** 1 Social, Genetic, and Developmental Psychiatry Centre (SGDP), Institute of Psychiatry, Psychology & Neuroscience, King’s College London, London, UK; 2 Division of Psychology and Language Sciences, University College London, London, UK

## Abstract

Recent studies highlight the role of excessive mind wandering in attention-deficit/hyperactivity disorder (ADHD) and its association with impairment. We believe assessing mind wandering could be especially relevant to individuals, including many females, who present with less externalising manifestations of ADHD. Using a new measure based on ADHD patient reports, the Mind Excessively Wandering Scale (MEWS), we previously found adults with ADHD had elevated levels of mind wandering that contributed to impairment independently of core ADHD symptoms. Using data from an online general population survey, the current study assessed the factor-structure, reliability, validity and measurement invariance of the MEWS. We also investigated sex differences in mind wandering, as well as ADHD symptoms, impairment and wellbeing in those with and without ADHD. The MEWS had a unidimensional structure, was invariant across sex, age and ADHD status, and accounted for unique variance in impairment and wellbeing beyond core ADHD symptoms. Among those with ADHD, we found no evidence for sex differences in mind wandering and among those without ADHD males had higher scores. We also found similar levels of hyperactivity/impulsivity, emotional lability, and impairment in males and females with ADHD, but males reported greater inattention and lower wellbeing. Results suggest the MEWS is a reliable and valid instrument measuring the same construct across sex, age and ADHD status, which could aid diagnosis and monitoring of outcomes.

## Introduction

Given the extensive evidence that ADHD is not confined to childhood and also occurs in adulthood, identifying measures to aid diagnostic assessment and monitoring of treatment outcomes in this age group is of considerable interest to research and clinical practice^1^. Although ADHD diagnosis is made based on the presence of core symptoms of inattention and hyperactivity/impulsivity^2^, other characteristic features, such as subjective reports of excessive mind wandering could aid the diagnostic process and/or monitoring of treatment outcomes^3^. Mind wandering is conceptualised as periods in time when attention and the contents of thoughts shift away from external sources and/or ongoing tasks, to unrelated internal thoughts or feelings^4^. Excessive mind wandering has been linked to impairment in ADHD^3,5–9^ and could reflect a core underlying symptom^10^.

In contrast to the core ADHD symptoms, mind wandering reflects internal thought processes, as opposed to directly observable behaviours. Excessive mind wandering may be particularly relevant to the assessment of ADHD in adolescence and adulthood when self-report plays a larger role in the diagnostic process. It may also be more difficult for individuals to reflect upon the behavioural symptoms that are currently used in the diagnostic criteria, while they may be better able to report on internal thought processes. Furthermore, the traditional symptoms of ADHD may not be obvious in people who have developed good adaptive skills that mask the behavioural symptoms of ADHD. Assessing mind wandering in ADHD could be especially relevant to individuals, including many females, who present with less externalising manifestations of ADHD. Thus, investigation of sex differences in mind wandering is especially pertinent as it does not depend on behavioural adaption to the same degree as traditional ADHD measures.

We recently developed and validated a new rating scale reflecting excessive mind wandering in ADHD: the Mind Excessively Wandering Scale (MEWS)^3^. The scale demonstrated a unidimensional structure with good internal consistency, and high sensitivity and specificity to discriminate between ADHD cases and controls, behaving in a comparable way to existing ratings scales of ADHD symptoms used in clinical practice. Adult ADHD cases showed elevated levels of mind wandering that contributed to impairment independently of the core ADHD symptom domains. Thus, excessive, uncontrolled mind wandering appears to be a common co-occurring feature of adult ADHD with specific implications for impairment in daily-life^3^.

The current study sought to further validate the MEWS in a large adult population sample, including those who report a diagnosis of ADHD, and assess measurement invariance of the scale across sex, ADHD diagnostic status, and age. Measurement invariance examines whether a scale captures fundamentally the same processes and construct across groups. Comparisons of group means for any scale is based on the assumption that the scale is measurement invariant across groups being compared, and a lack of invariance can render between-group comparisons meaningless and lead to incorrect interpretation of differences^11,12^. Between-group differences (e.g., ADHD cases vs controls) should not be analysed unless measurement invariance is held across the groups, yet this is rarely tested in empirical studies of psychiatric disorders^12^.

Given the well documented sex differences in ADHD^13,14^, and persistence of ADHD across the lifespan, it is important that the scale is also measurement invariant across sex and age. Further, as previous studies show mixed results with regard to whether males and females are affected differently by ADHD in adulthood^15–20^, we examined sex differences within those with and without self-reported ADHD with regard to mind wandering, as well as inattention, hyperactivity/impulsivity, emotional lability, impairment and wellbeing. Given that sex differences in adult ADHD have received much less attention in the literature compared to childhood^14,15,21^, there is a clear need for additional research. Furthermore, given that mind wandering may account for unique variance in functional impairment beyond core ADHD symptoms, and impairment is the most frequent reason for referral, investigating sex differences in mind wandering is clinically relevant.

## Methods

### Sample

This study uses data from a large online survey of individuals from the general population. The survey was advertised through King’s College London research recruitment page, ADHD user-group and information websites, and social media. The only exclusion criterion was participants could not be below 16 years of age. Data from 1484 participants (425 males, 1059 females) who fully completed the MEWS were used for analysis in the current study.

Participants were aged between 16–83 years (*M* = 34.80, SD = 13.55) and were categorised into age groups as follows: 1) 16–23 years (*n* = 382), 2) 24–30 years (*n* = 360), 3) 31–45 years (*n* = 365), and 4) 46 + years (*n* = 372). Those with and without full MEWS data did not differ significantly for sex (χ^2^(1) = 1.05, *p* = 0.31) or age group (χ^2^(3) = 5.74, *p* = 0.13). Participants who endorsed a childhood or adulthood diagnosis of ADHD based on self-report were included in the ADHD group (*n* = 198: 76 males, 122 females) and those reporting no diagnosis or ‘not sure’ (*n* = 59) were included in the non-diagnosed ADHD group (*n* = 1181).

### Ethical approval

Ethical approval was granted by the East of England–Cambridgeshire and Hertfordshire REC (ref:16/EE/0226). The study was carried out in compliance with the Helsinki Declaration of 1975, as revised in 2008. Participants provided informed consent online prior to completing the survey.

### Materials

#### Mind Wandering

Excessive mind wandering was measured using The Mind Excessively Wandering Scale (MEWS)^3^. While the MEWS was initially developed as a 15-item scale, previous psychometric evaluation and validation found 3 items had low factor loadings and that shortening the scale to 12-items did not reduce its sensitivity or specificity, so we use the 12-item measure.

To assess validation of the MEWS, unintentional (spontaneous) and intentional (deliberate) mind wandering were also assessed with the Mind Wandering Spontaneous (MW-S) and Mind Wandering Deliberate (MW-D) self-report scales^22^. Previously it has been shown that spontaneous, but not deliberate mind wandering is associated with ADHD^23^.

#### ADHD symptoms, functional impairment, emotional lability and positive mental health/wellbeing

ADHD symptoms were assessed using the self-rated Barkley Adult ADHD Rating Scale which consists of 18 items that closely parallel the DSM-5 symptom criteria for ADHD; 9 items pertain to inattention and 9 to hyperactivity/impulsivity^24^. The Barkley Current Behaviour Scale Self-report was used to measure the degree to which a participant’s inattention and hyperactivity/impulsivity symptoms cause problems for them in major life domains (e.g., family, work, education, social, life-skills, relationships, money, driving, recreation, and daily responsibilities)^24^. Emotional lability was assessed with the Affective Reactivity Index self-report measure of irritability^25^, and the Mental Health Continuum-Short Form assessed wellbeing (emotional, psychological, and social)^26^.

Further detail on the measures, including Cronbach’s alpha, are provided in Supplementary Table S1.

### Statistical analysis

Statistical analyses were carried out using MPlus^27^ and Stata^28^.

#### Factor Analysis

First, we performed item factor analysis to identify the dimensionality of the MEWS, using exploratory factor analysis (EFA) and confirmatory factor analysis (CFA). We used a random number algorithm to split the sample into two halves, which did not significantly differ for sex (χ^2^(1) = 0.95, *p* = 0.33) or age group (χ^2^(3) = 2.64, *p* = 0.45); EFA was carried out on the first half (*n* = 742) and CFA (*n* = 742) on the second. EFA (oblimin rotation) and CFA were carried out using a structural equation modelling framework and the Robust Weighted Least Squares (WLSMV) estimator, since this does not make distributional assumptions and is more appropriate for use with categorical variables^29^. Goodness of fit was assessed based on the following^30^: standardised mean square residual (SMSR), root mean square error of approximation (RMSEA), Tucker-Lewis Index (TLI), and the comparative fit index (CFI). In line with recommendations, the following values were used as indicators of good-fit: SMSR < 0.08^29–32^, RMSEA < 0.06^30–32^, TLI values >0.9, and CFI close to 1^29,30,32^. Of note, although chi-square is the traditional fit index used to evaluate model fit, it is very sensitive to sample size and can be inflated in large samples^30,31,33^ and so this was not used as a goodness of fit (GFI) index for the CFA model.

#### Measurement Invariance

The total sample was used to test measurement invariance across sex, age, and ADHD status using multi-group CFA (MGCFA) for categorical variables (sex and ADHD status) and multiple indicators multiple causes (MIMIC) models for continuous variables (age). MGCFA is tested in a sequential/hierarchical manner where constraints are consecutively added to the model. The first step involves running an unconstrained model for all groups combined to test for configural invariance (whether the same factor structure is observed between-groups). This is followed by a series of constrained models where parameters (factor-loadings and thresholds) are constrained to be equal across groups. Metric invariance refers to when factor-loadings are equivalent across groups, and scalar invariance refers to when the factor-loadings and item-thresholds are equivalent across the groups. Certain parameters are fixed for model identification (see Supplementary Table S2 for more detail). If the difference in fit indices between the model and the preceding, less constrained model, is ≤−0.01 for ΔCFI and ≤0.015 for ΔRMSEA, then we considered the corresponding level of measurement invariance was held^34–36^. If non-invariance was identified, modification indices were used to identify noninvariant items and remove the corresponding equality constraint (i.e., the parameter was freely estimated in each group). Then, if the fit indices were in line with the accepted cut-offs, partial invariance was held and the parameter remained unconstrained in the subsequent models. Of note, the nested chi-square difference test between two models (DIFFTEST) was not used due to its sensitivity to sample size (it has been noted that if sample size is greater than 200, any differences between groups indicated by the DIFFTEST are likely to be trivial and subsequent analyses can proceed)^37^.

MIMIC models allow covariates in the CFA model^29^ and as they do not split the sample by group can accommodate continuous covariates. If the covariate shows a significant direct effect on any of the individual scale items, this provides evidence of measurement non-invariance and the identified items are assumed to be affected by differential item functioning (DIF). However, if the magnitude of any direct effects is very small, then it is likely to have a trivial impact on the model^38^. In the baseline model, associations between the covariate and items are fixed to 0 and then modification indices are consulted, with modification indices >4 of the covariate on an item presenting DIF^35^. If the covariate shows an association with the latent structure (i.e., the factor), this provides evidence of population heterogeneity.

#### Reliability and Validity

To assess the reliability of the scale we estimated internal consistency using Cronbach’s alpha and examined item-total correlations. Convergent validity was assessed to provide an indication of the degree of relationship between the scale of interest (the MEWS) and other scales measuring similar entities, by calculating Pearson’s correlation coefficients between the MEWS and the MW-S, ADHD symptom scales, emotional lability, and impairment. To examine discriminant validity, providing an indication of whether two measures that should not be correlated are actually not related, we calculated Pearson’s correlation coefficients between the MEWS and MW-D and wellbeing. We then examined ADHD case-control differences and conducted receiver operating characteristic (ROC) analysis to examine the capacity of the scale to discriminate between those with and without ADHD.

We also carried out regression analysis in the ADHD group to assess whether mind wandering accounts for unique variance in impairment and wellbeing beyond that accounted for by the core ADHD symptoms, which would further emphasise the potential value of the scale. Specifically, a hierarchical regression was conducted with impairment/wellbeing summary scores as the dependent variable and inattention and hyperactivity/impulsivity summary scores entered in Step1, and MEWS summary scores entered in Step2.

#### Sex differences in those with and without self-reported ADHD

Sex differences across ADHD diagnostic status in mind wandering, as well as ADHD symptoms, emotional lability, impairment, and wellbeing, were tested using linear regression models adjusted for age. Cohen’s d was used as an indication of effect size where: d ≥ 0.20 is a small effect, d ≥ 0.50 a medium effect, and d ≥ 0.80 a large effect.

## Results

### Factor Analysis

All 12-items in the scale were included in the EFA model. The sample correlation matrix produced 1 eigenvalue >1 (8.55), in line with the scree plot (Supplementary Fig. S1). The next largest eigenvalue was 0.89. Goodness of fit indices for the 1-factor model were: SMSR = 0.06, RMSEA = 0.13 [95% CI: 0.12, 0.14], TLI = 0.97, CFI = 0.97. Next, we entered the 12-items into a CFA model specifying a 1-factor solution. Each of the 12-items demonstrated a high loading on to the single hypothesised factor (>0.75) (Table 1.). Three out of four fit indices (SMSR = 0.06, RMSEA = 0.15 [0.14, 0.16], TLI = 0.96, CFI = 0.96) were indicative of good/acceptable model fit; only the RMSEA was higher than the recommended cut-off.

**Table 1:** Standardised factor loadings for the 12-items for EFA (with oblimin rotation) and CFA and across sex and ADHD status, and Cronbach’s alpha for the scale.

Item		Standardised Factor Loadings					
		EFA (*n* = 742)	CFA (*n* = 742)	Males (*n* = 425)	Females (*n* = 1059)	ADHD (*n* = 198)	No ADHD (*n* = 1181)
1.	I have difficulty controlling my thoughts	0.83	0.80	0.83	0.81	0.75	0.80
2.	I find it hard to switch my thoughts off	0.82	0.80	0.82	0.81	0.80	0.79
3.	I have two or more different thoughts going on at the same time	0.74	0.76	0.69	0.78	0.73	0.72
4.	My thoughts are disorganised and ‘all over the place’	0.85	0.88	0.85	0.87	0.80	0.84
5.	My thoughts are ‘on the go’ all the time	0.84	0.84	0.88	0.83	0.89	0.82
6.	I experience ceaseless mental activity	0.86	0.82	0.85	0.84	0.82	0.82
7.	I find it difficult to think about one thing without another thought entering my mind	0.88	0.84	0.87	0.86	0.83	0.85
8.	I find my thoughts are distracting and prevent me from focusing on what I am doing	0.87	0.88	0.89	0.87	0.80	0.86
9.	I have difficulty slowing my thoughts down and focusing on one thing at a time	0.91	0.91	0.90	0.91	0.82	0.90
10	I find it difficult to think clearly, as if my mind is in a fog	0.78	0.79	0.78	0.78	0.65	0.77
11.	I find myself flitting back and forth between different thoughts	0.88	0.83	0.83	0.86	0.76	0.84
12.	I can only focus my thoughts on one thing at a time with considerable effort	0.79	0.79	0.80	0.79	0.54	0.78
Internal consistency	Cronbach’s Alpha (α)	0.95		0.95	0.95	0.91	0.94

### Measurement Invariance

Table 1 shows standardised factor loadings for the MEWS items across sex and ADHD status. Table 2 summarises the change in goodness of fit indices for measurement invariance (assessed with MGCFA) across sex and ADHD status.

**Table 2:** Multi-group CFA models for measurement invariance across sex and ADHD diagnostic status for the MEWS.

	Measurement invariance model^a^ (constraints)	CFI	ΔCFI	RMSEA (95% CI)	ΔRMSEA
Sex	Configural (no equality constraints)	0.968	—	0.141 (0.135, 0.147)	—
	Metric (factor loadings)	0.969	0.001	0.132 (0.127, 0.138)	−0.009
	Scalar (factor loadings and thresholds)	0.970	0.001	0.120 (0.115, 0.125)	−0.012
ADHD	Configural	0.960	—	0.138 (0.132, 0.144)	—
	Metric	0.964	0.004	0.125 (0.120, 0.131)	−0.013
	Scalar	0.964	0.000	0.113 (0.108, 0.119)	−0.012

^a^See Supplementary Table S2 for further detail on the constraints applied to each model.

#### Sex

The configural model showed acceptable model fit according to TLI and CFI (TLI = 0.961, CFI = 0.968), although RMSEA was higher than the recommended cut-off (RMSEA = 0.141[0.135, 0.147]). Thus, model fit indices were similar to those for the CFA carried out above, with two out of three fit indices reaching acceptable levels, implying the factor structure is equivalent between males and females. Metric and scalar invariance both held in relation to the ΔCFI and ΔRMSEA.

#### ADHD status

The configural model showed acceptable model fit according to TLI and CFI (TLI = 0.951, CFI = 0.960), although RMSEA was higher than the recommended cut-off (RMSEA = 0.138 [0.132, 0.144]). Thus, model fit indices were similar to the CFA results, with two out of three fit indices reaching acceptable levels, implying the factor structure is equivalent between those with and without a self-reported ADHD diagnosis. Both metric and scalar invariance held as assessed by ΔCFI and ΔRMSEA.

Thus, the MEWS showed full measurement invariance across sex and ADHD status at the scalar level, and we are satisfied that there is no substantial measurement bias.

#### Age

MIMIC analysis testing the direct effect of age on individual items suggested DIF for Item 12 (“*I can only focus my thoughts on one thing at a time with considerable effort*”) as a function of age. Assuming equivalent levels of mind wandering, increasing age was associated with increased scores for this item (direct-effect = 0.01, *p* < 0.001); however, the magnitude of the direct effect is small. Further, consideration should be given to the fact that Item 12 appeared last in the scale and the effect could be an artefact of this. Results therefore indicate that age is not a concern regarding measurement non-invariance. Regarding population heterogeneity, the MIMIC model suggested that mind wandering increased with age.

Thus, MGCFA and MIMIC indicate the MEWS is measurement invariant across sex, ADHD, and age.

### Reliability and Validity

Internal consistency for the 12-item scale was high for the complete sample (α = 0.95), with no improvement in the reliability index gained by omitting items. Item-total correlations were all above 0.69. Similar results were found across sex and ADHD status. For males and females α was 0.95, with no improvement in the reliability index gained by omitting items, and item-total correlations all >0.64. Alpha was also high in those with ADHD (α = 0.91) and those without (α = 0.94), with no improvement in the reliability index gained by omitting items, and item-total correlations >0.48 in those with ADHD and >0.66 in those without ADHD.

Demonstrating convergent validity, the MEWS correlated moderately-to-strongly with spontaneous mind wandering (MW-S *r* = 0.76, *p* < 0.001), ADHD symptoms (inattention *r* = 0.76, *p* < 0.001; hyperactivity/impulsivity *r* = 0.71, *p* < 0.001), emotional lability (*r* = 0.44, *p* < 0.001), and impairment (*r* = 0.74, *p* < 0.001) (Table 3). It also demonstrated discriminant validity, with a weak, non-significant correlation with deliberate mind wandering (MW-D *r* = 0.05, *p* = 0.06) and a negative relationship with wellbeing (*r* = −0.41, *p* < 0.001).

**Table 3:** Correlations between excessive mind wandering scores and rating-scale measures of spontaneous and deliberate mind wandering, inattention hyperactivity/impulsivity, emotional lability, impairment, and wellbeing.

	MEWS	MW-D	MW-S	INN	HI	EL	IMP
MEWS	—	—	—	—	—	—	—
MW-D	0.05	—	.	—	—	—	—
MW-S	**0.76**	**0.19**	—	—	—	—	—
INN	**0.76**	0.03	**0.67**	—	—	—	—
HI	**0.71**	0.03	**0.58**	**0.72**	—	—	—
EL	**0.44**	−0.04	**0.35**	**0.41**	**0.43**	—	—
IMP	**0.74**	0.01	**0.63**	**0.84**	**0.68**	**0.46**	—
WB	**−0.41**	**0.10**	**−0.30**	**−0.40**	**−0.21**	**−0.37**	**−0.46**

*Note*. MEWS = Mind Excessively Wandering Scale; MW-S = Mind Wandering Spontaneous; MW-D = Mind Wandering Deliberate; INN = inattention; HI = hyperactivity/impulsivity; EL = emotional lability; IMP = impairment; WB = wellbeing. Statistically significant correlations are presented in bold: significant at p < 0.001.

We found elevated levels of mind wandering as measured by the MEWS in those with a self-reported diagnosis of ADHD compared to those without ADHD (*p* < 0.001, *d* = −1.17), with the same pattern of findings for measures of spontaneous mind wandering (MW-S) (*p* < 0.001, *d* = −0.98), inattention (*p* < 0.001, *d* = −1.54), hyperactivity/impulsivity (*p* < 0.001, *d* = −1.42), emotional lability (*p* < 0.001, *d* = −0.45), and impairment (*p* < 0.001, *d* = −1.47). We also found higher scores for wellbeing in those without ADHD compared to ADHD cases (*p* < 0.001, *d* = 0.35) (see Supplementary Table S3 for mean scores across subscales). ROC analysis examined the capacity of the MEWS to discriminate between those with and without ADHD. Area under the curve (AUC) was 0.81 (95% CI: 0.78, 0.84, *p* < 0.001); the closer the value to 1, the better the discriminant capacity of the measure, and so this indicates that in the current sample including individuals with self-reported ADHD the MEWS has good discriminant capacity. This was comparable to the inattention (AUC = 0.86, 95% CI: 0.84, 0.89) and hyperactivity/impulsivity rating scales in the sample (AUC = 0.83, 95% CI: 0.80, 0.86).

Regression analysis in the ADHD group examining if mind wandering (measured by the MEWS) accounts for unique variance in impairment beyond core ADHD symptoms found that inattention and hyperactivity/impulsivity accounted for 53.4% of the variability in impairment (*R*^2^ = 0.53). The addition of mind wandering in the model led to a significant increase in the variability accounted for by the model (*R*^2^Δ = 0.02), with an increase to 55.3%, *F*Δ(1,194) = 8.34, *p* = 0.004. This indicates that mind wandering is having a small but significant effect beyond that of inattention and hyperactivity/impulsivity. Inattention was the most strongly associated (β = 0.56), followed by mind wandering (β = 0.19) and hyperactivity/impulsivity (β = 0.08). Only inattention and mind wandering were significantly associated with impairment in the model (*p* < 0.001 and *p* = 0.004, respectively).

Similar findings were found for the measure of wellbeing. Within those with self-reported ADHD, mind wandering accounted for unique variance in total wellbeing beyond core ADHD symptoms. Inattention and hyperactivity/impulsivity accounted for 10% of the variance in wellbeing (*R*^2^ = 0.10). The addition of mind wandering as a predictor led to a significant increase in the variability accounted for by the model (*R*^2^Δ = 0.04), with an increase to 15%, *F*Δ(1,194) = 9.29, *p* = 0.003. Mind wandering was the most strongly associated (β = −0.28, *p* = 0.003), followed by inattention (β = −0.25, *p* = 0.009) and hyperactivity/impulsivity (β = 0.24, *p* = 0.005). This implies that excessive mind wandering in ADHD is having a small but significant independent negative effect on wellbeing beyond that accounted for by the core ADHD symptoms.

### Sex differences in those with and without self-reported ADHD

Overall (irrelevant of diagnostic status), males had significantly higher scores across all scales (*p* range < 0.001 to 0.004, *d* range 0.12 to 0.44), except greater wellbeing reported by females (*p* < 0.001, *d* = −0.25) and no significant difference in emotional lability (*p* = 0.17) (Supplementary Table S3). In those with self-reported ADHD, males and females showed similar symptom scores, except males with ADHD reported significantly greater levels of inattention (*p* = 0.045, *d* = 0.32) and lower wellbeing than females (*p* = 0.01, *d* = −0.37) (Table 4). Among those without ADHD, males reported significantly greater symptom levels across all variables (*p* range < 0.001 to 0.04, *d* range 0.12 to 0.41) and lower wellbeing than females (*p* = 0.008, *d* = −0.20).

**Table 4:** Mean scores (SD) for the study subscales comparing males and females with and without ADHD*.

	ADHD				No ADHD			
	Males (n = 76)	Females (n = 122)	*p*	Cohen’s d (95% CI)	Males (n = 319)	Females (n = 862)	*p*	Cohen’s d (95% CI)
MEWS	26.34 (6.57)	25.38 (7.75)	0.44	0.13 (−0.16, 0.42)	16.90 (9.98)	15.36 (8.53)	**0.01**	0.18 (0.05, 0.31)
MW-S	23.41 (3.44)	23.47 (4.60)	0.78	−0.01 (−0.30, 0.27)	18.99 (5.39)	17.96 (5.50)	**0.006**	0.19 (0.06, 0.32)
MW-D	17.39 (7.07)	17.16 (6.35)	0.78	0.04 (−0.25, 0.32)	18.16 (5.58)	17.47 (5.72)	**0.008**	0.12 (−0.01, 0.25)
INN	19.92 (4.27)	18.30 (5.48)	**0.045**	0.32 (0.03, 0.61)	11.34 (6.67)	8.80 (6.02)	**<0.001**	0.41 (0.28, 0.54)
HI	15.70 (5.10)	15.31 (6.43)	0.75	0.06 (−0.22, 0.35)	8.86 (5.46)	7.74 (4.92)	**0.003**	0.22 (0.09, 0.35)
EL	3.63 (2.91)	4.29 (3.17)	0.13	−0.21 (−0.50, 0.07)	3.02 (3.11)	2.65 (2.69)	**0.04**	0.13 (0.002, 0.26)
IMP	2.02 (0.55)	1.90 (0.63)	0.23	0.20 (−0.09, 0.49)	1.08 (0.72)	0.86 (0.70)	**<0.001**	0.31 (0.18, 0.44)
WB	42.42 (13.77)	47.26 (12.92)	**0.01**	−0.37 (−0.65, −0.08)	48.24 (13.89)	51.09 (14.45)	**0.008**	−0.20 (−0.33, −0.07)

*Note*. MEWS = Mind Excessively Wandering Scale; MW-S = Mind Wandering Spontaneous; MW-D = Mind Wandering Deliberate; INN = inattention; HI = hyperactivity/impulsivity; EL = emotional lability; IMP = impairment; WB = wellbeing. Statistical analysis adjusted for age. Statistically significant findings are presented in bold. *1379 participants answered the question regarding a previous diagnosis of ADHD.

## Discussion

We validated the Mind Excessively Wandering Scale (MEWS) using a large population sample. The scale showed measurement invariance across sex, age and ADHD diagnostic status, suggesting the MEWS is a reliable and valid instrument measuring the same construct across the studied groups. Among those with ADHD, we found no evidence for sex differences in mind wandering, nor were there sex differences in levels of hyperactivity/impulsivity, emotional lability, or impairment. However, males with ADHD reported higher levels of inattention and lower wellbeing than females with ADHD. Among individuals without ADHD, males had higher scores across all ADHD related scales and reported significantly lower wellbeing than females.

In the current study, analysis involved a series of factor analysis models. EFA suggested that a 1-factor structure is appropriate for the MEWS. Model fit indices for the CFA model were acceptable, with three out of four fit indices suggesting acceptable fit for the unidimensional structure. The RMSEA exceeded the recommended cut-off, however this does not mean that one should automatically disregard the model^39^. It can be difficult to achieve good fit in large samples^31,40^ and fit indices should be interpreted with this in mind. Further, there is also debate regarding model fit indices and the ‘rules of thumb’, with a general consensus that strictly adhering to recommended cut-offs can lead to incorrectly rejecting an acceptable model (i.e., a Type I error)^30^ and that allowing model fit to drive the research process moves away from the theory-testing purpose of structural equation modelling^30,31^. Thus, we believe the results support the MEWS as measuring one unified construct of excessive mind wandering.

This study is the first to test measurement invariance of the MEWS, which is important to establish for any new measure, and extends our previous findings. The MEWS had full scalar invariance across sex and ADHD diagnostic status. No sex differences were observed at the level of factor loadings or thresholds, indicating scalar invariance across sex in the total sample. This attests that the same latent construct of mind wandering is related to the items and their thresholds similarly for males and females. Measurement invariance also held in those with and without a self-reported diagnosis of ADHD (i.e., scalar invariance held). Thus, comparisons of mean scores across these groups is appropriate and meaningful, and any between group differences observed can be reliably interpreted as true differences in the latent construct of mind wandering. We also found each scale item functioned similarly across age, but that increasing age may be associated with higher levels of mind wandering (i.e., the latent construct), so age should be considered as an important covariate in analyses of mind wandering.

Reliability and validity analysis showed the 12-item scale had high internal consistency in the total sample, as well as in males and females and those with and without self-reported ADHD. The MEWS demonstrated convergent validity, with moderate to strong correlations found between the MEWS and measures of core ADHD symptoms, impairment, and wellbeing (negative correlation). The MEWS showed strong correlation with another existing measure of spontaneous mind wandering but not with deliberate mind wandering, supporting the MEWS as specifically reflecting spontaneous mind wandering. Of note, the correlation between the MEWS and emotional lability was lower than demonstrated in our previous study, which may be due to different measures of emotional lability being employed in the studies. Regarding discriminant validity, both ROC analysis and examination of mean differences showed the MEWS distinguished between those with and without self-reported ADHD. Importantly, in the current sample the ROC analysis using the MEWS produced results comparable to those of the core ADHD symptom dimensions.

The National Collaborating Centre for Mental Health^41^ emphasise the importance of linking symptoms to impairment, which may be a better measure of identifying those who would benefit from treatment rather than somewhat arbitrary symptom counts. Replicating our previous findings, among adults with ADHD, mind wandering accounted for unique variance in impairment above that of core ADHD symptoms of inattention and hyperactivity/impulsivity. The ability for the MEWS to account for unique variance beyond core ADHD symptoms demonstrates the value of the measure and its potential clinical utility. Similar findings were also found for wellbeing, further emphasising the value of the scale. Thus, findings indicate that mind wandering is associated with both increased impairment and reduced wellbeing even after accounting for levels of inattention and hyperactivity/impulsivity.

Examination of sex differences showed that among those without ADHD males reported greater levels of mind wandering compared to females, but among those with ADHD, males and females reported similar levels. A similar pattern was found for inattention and hyperactivity/impulsivity, with the exception that among those with ADHD males reported more inattention than females. This is in line with recent findings amongst youth^42^ showing higher ADHD symptoms in males than females in the general population, but similar symptom severity amongst those with a diagnosis, except for higher inattention in males. To our knowledge this is the first investigation of sex differences in those with and without ADHD in a population-based sample of adults, and we found that the pattern of sex differences for the behavioural ADHD symptoms in adults with and without ADHD is also reflected in the more internalised/subjective experience of excessive mind wandering (i.e., similar severity in males and females with a diagnosis and greater scores in males vs females without a diagnosis).

Regarding impairment, males without ADHD reported greater impairment than females, but similar levels were reported among those with a self-reported ADHD diagnosis. Additionally, despite similar symptom levels and impairment in males and females with ADHD, females with ADHD reported significantly greater wellbeing, potentially indicating differing perceptions of behaviour in males and females with ADHD. In youth, studies show parents may under-rate ADHD symptoms and impairment in girls with ADHD compared to boys, suggesting sex-specific biases in perceptions of behaviour^43–45^. It is possible that differing perceptions of behaviours and impairment in males and females also occurs in adulthood. This requires further investigation.

A strength of the current study is the large sample size including a large age range and representation of females, both of which are often a limitation of studies in the ADHD literature. However, some limitations should be considered. First, despite the online survey being a general population sample, it is not necessarily representative of the general population, but of those who chose to participate in an online survey. Our sample included significantly more females than males suggesting that females may be more willing to participate in such studies. Moreover, it may mean that the findings regarding sex differences are not generalisable. Further studies should examine if results are replicated across other populations and those with a more equal sex balance. Second, our definition of ADHD was based on self-report and not clinical diagnosis data. However, this approach has previously been employed in a survey design^46^, and was adopted in the most recent large-scale genome-wide association study of ADHD using 23 and me^47^. In addition, our findings replicate those of our previous study that included a clinical sample of adults with ADHD^3^.

Enhancing our understanding of the broader range of symptoms or problems associated with ADHD and the phenomenology that underlies ADHD symptomatology has the potential to aid diagnosis and inform targets for interventions. Valid and reliable assessment of disorders is a prerequisite for clinical treatment and intervention, and the MEWS has been shown to be a reliable and valid measure of mind wandering in ADHD that discriminates between those with and without ADHD, with satisfactory sensitivity and specificity, and specific association with functional impairment. Finding measures to assess adult ADHD, a disorder primarily defined in behavioural terms, is of value, and mind wandering could be more useful when self-report is possible as it is the experience of the internal mental states rather than observed behaviours. The MEWS shows promise as a brief screening tool in the general population and could be a potential target for monitoring of treatment effects.

The replicated finding that mind wandering had an independent effect on impairment beyond that of core ADHD symptoms of inattention and hyperactivity/impulsivity, indicates that symptoms of mind wandering are strongly linked to impairment and so may be an important target for therapeutic interventions. Further studies are needed to establish such interventions, but mindfulness training could be an area of future emphasis.

## Supplementary information

Supplementary Online Content

## Data Availability

The dataset analysed during the current study is available from the corresponding author upon reasonable request.
