# Supplementary material for: Evaluating a scale of excessive mind wandering among males and females with and without attention-deficit/hyperactivity disorder from a population sample
Source: Sci Rep. 2019 Feb 28;9:3071. doi: 10.1038/s41598-019-39227-w (PMC6395591; doi:10.1038/s41598-019-39227-w)
Supplement: Supplementary file 1 — Supplementary Online Content [file 41598_2019_39227_MOESM1_ESM.pdf]

Supplementary Online Content

Mowlem, F.D., Agnew-Blais, J., Pingault, JB, & Asherson, P. Evaluating a scale of excessive mind wandering among males and females with and without ADHD from a population sample. *Scientific Reports*

Correspondence: Florence Mowlem, SGDP Centre, Institute of Psychiatry, Psychology & Neuroscience, DeCrespigny Park, Denmark Hill, London, SE5 8AF; florence.d.mowlem@kcl.ac.uk

Supplementary Table S1. Details of the study measures

Supplementary Table S2. Details of the constraints applied to the 3 models used to test measurement invariance

Supplementary Table S3. Mean scores (SD) for the study subscales stratified by sex and self-reported ADHD diagnostic status, comparing males to females, and those with ADHD to those without ADHD

Supplementary Fig S1. Scree plot for EFA

Supplementary Table S1. Details of the study measures

Measure	Length	Scoring	Maximum score	Cronbach's alpha
The Mind Excessively Wandering Scale (MEWS) [1]	12 items	4-point likert scale<br><br>*Not at all or rarely* [0], *Some of the time* [1], Most *of the time* [2], N*early all of the time or constantly* [3]	36	.95
The Mind Wandering Spontaneous Scale (MW-S) [2]	4 items	7-point likert scale<br><br>*Rarely* [1] to *A lot* [7], or *Almost never* [1] to *Almost always* [7]	28	.86
The Mind Wandering Deliberate Scale (MW-D) [2]	4 items	7-point likert scale<br><br>*Rarely* [1] to *A lot* [7]*, or Not at all true* [1] *to Very true* [7]	28	.84
Barkley Adult ADHD Rating Scale [3]	18 items<br>(9 items for inattention, 9 items for hyperactivity/impulsivity)	4-point likert scale<br><br>*Never or Rarely* [0], *Sometimes* [1], *Often* [2], *Very Often* [3]	54 (27 per subscale)	Entire scale: .93<br>Inattention: .92<br>Hyperactivity: .86
Barkley Current Behaviour Scale Self-report [3]	10 items	4-point likert scale<br><br>*Never or Rarely* [0], *Sometimes* [1], *Often* [2], *Very Often* [3]<br><br>A '*Not Applicable*' option is available, which should be selected if the item refers to an area that is not part of their life (e.g. they do not drive), as opposed to not experiencing impairment in that domain.	30	.93
The Affective Reactivity Index (ARI) [4]	7 items<br>(6 symptom items, 1 impairment item)	3-point likert scale<br><br>*Not True* [0], *Somewhat True* [1], *Certainly True* [2]	12	.86
The Mental Health Continuum-Short Form (MHC-SF) [5]	14 items<br>(6 items for psychological wellbeing, 5 items for social wellbeing, 3 items for emotional wellbeing)	6-point likert scale<br><br>*Never* [0], *Once or twice* [1], *About once a week* [2], *About 2 or 3 times a week* [3], *Almost every day* [4], *Every day* [5]	70	.92

Supplementary Table S2. Details of the constraints applied to the 3 models used to test measurement invariance

	**Loadings**	**Thresholds**	**Item residual variances**	**Factor variance**	**Factor mean**
**Configural**	Free to vary in both groups (one factor loading fixed at 1 for scaling purposes)	Free to vary in both groups	Fixed at 1	Free to vary in both groups	Fixed at 0 (for identification purposes)
**Metric**	Equal across groups	Free (first threshold of each item is held equal across groups, and second threshold of the item used to set the metric of the factor is held equal across groups)	Fixed at 1 in first group and free in the other	Free to vary in both groups	Fixed at 0 in first group and free in the other
**Scalar**	Equal across groups	Equal across groups	Fixed at 1 in first group and free in the other	Free to vary in both groups	Fixed at 0 in first group and free in the other

Supplementary Table S3. Mean scores (SD) for the study subscales stratified by sex and self-reported ADHD diagnostic status, comparing males to females, and those without ADHD to those with ADHD

	Whole sample (*n*=1379-1484)	Females (*n*=984-1059)	Males (*n*=395-425)	Males vs Female		ADHD (*n*=198)	No ADHD (*n*=1180-1181)	No ADHD vs ADHD	
	M (SD)	M (SD)	M (SD)	*p*	Cohen's d (95% CI)	M (SD)	M (SD)	*p*	Cohen's d (95% CI)
MEWS	17.16 (9.21)	16.57 (9.11)	18.65 (9.29)	**.001**	.23 (.11, .34)	25.75 (7.32)	15.77 (8.68)	**<.001**	-1.17 (-1.33, -1.02)
MW-S	18.96 (5.66)	18.61 (5.75)	19.84 (5.34)	**.002**	.23 (.10, .33)	23.44 (4.18)	18.24 (5.49)	**<.001**	-.98 (-1.13, -.82)
MW-D	17.75 (5.88)	17.25 (5.87)	17.95 (5.87)	**.004**	.12 (.01, .23)	17.25 (6.62)	17.65 (5.69)	1.0	.07 (-.08, .22)
INN	10.84 (6.97)	9.97 (6.71)	12.98 (7.12)	**<.001**	.44 (.33, .55)	18.92 (5.10)	9.49 (6.30)	**<.001**	-1.54 (-1.70, -1.37)
HI	9.08 (5.86)	8.66 (5.70)	10.15 (6.10)	**<.001**	.26 (.14, .37)	15.46 (5.94)	8.04 (5.09)	**<.001**	-1.42 (-1.58, -1.26)
EL	3.00 (2.93)	2.93 (2.86)	3.19 (3.10)	.17	.09 (-.02, .20)	4.04 (3.08)	2.75 (2.82)	**<.001**	-.45 (-.60, -.30)
IMP	1.06 (0.79)	0.99 (0.77)	1.25 (0.79)	**<.001**	.34 (.22, .45)	1.95 (0.60)	0.92 (0.72)	**<.001**	-1.47 (-1.63, -1.30)
WB	49.61 (14.32)	50.61 (14.31)	47.12 (14.04)	**<.001**	-.25 (-.36, -.13)	45.40 (13.42)	50.32 (14.35)	**<.001**	.35 (.19, .50)

Note. MEWS = Mind Excessively Wandering Scale; MW-S = Mind Wandering Spontaneous; MW-D = Mind Wandering Deliberate; INN = inattention; HI = hyperactivity/impulsivity; EL = emotional lability; IMP = impairment; WB = wellbeing.
Statistical analysis adjusted for age.
Statistically significant findings are presented in bold.

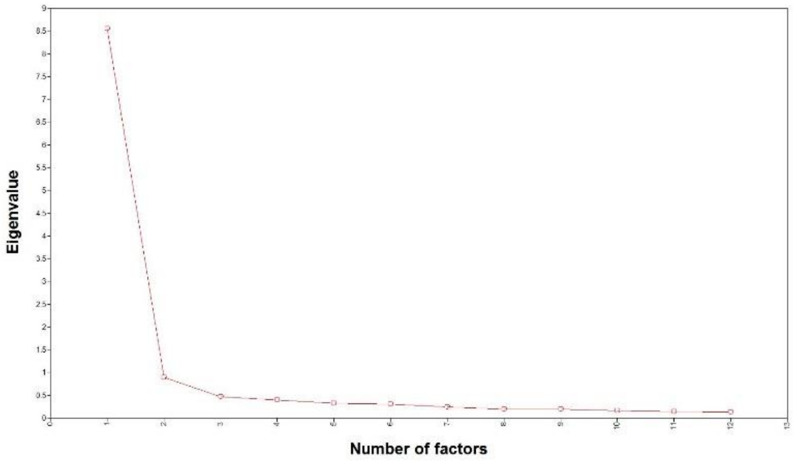

Supplementary Fig S1. Scree plot for EFA

References

1. Mowlem, F. D. *et al.* Validation of the Mind Excessively Wandering Scale and the Relationship of Mind Wandering to Impairment in Adult ADHD. *J. Atten. Disord.* (2016). doi:10.1177/1087054716651927

2. Carriere, J. S. a, Seli, P. & Smilek, D. Wandering in both mind and body: individual differences in mind wandering and inattention predict fidgeting. *Can. J. Exp. Psychol.* **67,** 19–31 (2013).

3. Barkley, R. A. & Murphy, K. *Attention-Deficit Hyperactivity Disorder: A Clinical Workbook*. (Guilford Press, 2006).

4. Stringaris, A. *et al.* The Affective Reactivity Index: A concise irritability scale for clinical and research settings. *J. Child Psychol. Psychiatry Allied Discip.* **53,** 1109–1117 (2012).

5. Keyes, C. L. M. *Brief description of the mental health continuum short form (MHC-SF)*. (2009).
